# Role of Scaffold Protein Proline-, Glutamic Acid-, and Leucine-Rich Protein 1 (PELP1) in the Modulation of Adrenocortical Cancer Cell Growth

**DOI:** 10.3390/cells6040042

**Published:** 2017-11-07

**Authors:** Arianna De Luca, Paola Avena, Rosa Sirianni, Adele Chimento, Francesco Fallo, Catia Pilon, Ivan Casaburi, Vincenzo Pezzi

**Affiliations:** 1 Cell and Applied Biology Laboratory, Department of Pharmacy, Health and Nutritional Sciences, University of Calabria, Arcavacata di Rende, 87036 Cosenza, Italy; ariannadl@hotmail.it (A.D.L.); paolaavena@libero.it (P.A.); rosa.sirianni@unical.it (R.S.); adechimento@libero.it (A.C.); 2 Clinica Medica 3, Department of Medicine-DIMED, University of Padova, 35128 Padova, Italy; Francesco.fallo@unipd.it (F.F.); catia.pilon@unipd.it (C.P.)

**Keywords:** adrenocortical carcinoma (ACC), proline glutamic acid and leucine rich protein 1 (PELP1), estrogen receptor α (ERα), insulin growth factor-1 receptor (IGF1R), insulin-like growth factor II (IGF-II)

## Abstract

PELP1 acts as an estrogen receptor (ER) coactivator that exerts an essential role in the ER’s functions. ER coregulators have a critical role in the progression and response to hormonal treatment of estrogen-dependent tumors. We previously demonstrated that, in adrenocortical carcinoma (ACC), ERα is upregulated and that estradiol activates the IGF-II/IGF1R signaling pathways defining the role of this functional cross-talk in H295R ACC cell proliferation. The aim of this study was to determine if PELP1 is expressed in ACC and may play a role in promoting the interaction between ERα and IGF1R allowing the activation of pathways important for ACC cell growth. The expression of PELP1 was detected by Western blot analysis in ACC tissues and in H295R cells. H295R cell proliferation decrease was assessed by A3-(4,5-Dimethylthiaoly)-2,5-diphenyltetrazolium bromide (MTT) assay and [3H] thymidine incorporation. PELP1 is expressed in ACC tissues and in H295R cells. Moreover, treatment of H295R with E2 or IGF-II induced a multiprotein complex formation consisting of PELP1, IGF1R, ERα, and Src that is involved in ERK1/2 rapid activation. PELP1/ER/IGF1R/c-Src complex identification as part of E2- and IGF-II-dependent signaling in ACC suggests PELP1 is a novel and more efficient potential target to reduce ACC growth.

## 1. Introduction

Adrenocortical carcinomas (ACC) are rare and highly aggressive tumors, associated with a very poor prognosis, mostly due to a high risk of recurrence and limited therapeutic options [1]. Complete surgical excision offers the best chance of long term survival but quite often, despite the complete resection, the tumor reinstates very recurrently [2]. The cause of adrenal cancer remains elusive, but studies in the past 10 years suggest genetic mutations in the adrenal gland lead to the initiation of a malignant tumor [3,4,5].

However, ACC is an extremely heterogeneous disease and the majority of currently published studies have analyzed only single pathways of signal transduction. It is becoming clear that ACC pathogenesis involves integration of signals and the interplay of downstream pathways. Among these, the IGF system and estrogen-dependent pathways appear to be of particular interest.

It has been demonstrated that the insulin-growth factor 2 (IGF-II) gene is strongly over-expressed in adrenocortical carcinomas, representing one of the most commonly identified mutations in ACC [6]. IGF-II indicates a reliable prognostic marker in this disease, suggesting that it can be used to identify patients with a high risk of recurrence [7,8,9]. In ACC cells, IGF-II induces mitogenic effects through interaction with the IGF1 receptor (IGF1R), resulting in the activation of the PI3K/AKT/mTOR cascade, as well as RAS/MAPK and the PLC/PKC pathways [10]. It is known that estrogens are produced by the enzyme aromatase using androgens as substrate, and we have already shown that ACC is characterized by aromatase overexpression [11]. Thus, we can speculate that, in ACC patients, despite the normal circulating estrogen levels, a higher local estrogen production can occur. The classical mechanisms of estrogen action are mainly mediated by two members of the nuclear receptor superfamily, the estrogen receptor (ER) α and β. In ACCs, we previously demonstrated that ERα is upregulated [11] and that estradiol enhances H295R cell proliferation [12]. Recently, we demonstrated both in vitro and in vivo the potential involvement of G-coupled-estrogen receptor (GPER) in H295R cell growth that was strongly inhibited by G1, a GPER agonist [13]. Furthermore, the growth inhibitory effect was also achieved using XCT790, the estrogen related receptor α (ERRα) inverse agonist [14]. These results suggest that estrogen signaling and related nuclear receptors are involved in ACC cell growth.

We also demonstrated that the existence of a functional interplay between the IGF-II/IGF1R system and estrogen signaling which turned out to be essential in controlling intracellular pathways crucial for ACC proliferation. In particular, our previous observations demonstrated that IGF-II caused ERα phosphorylation on serine 118 and serine 167 residues, activating ERα in a ligand independent manner and increasing cell proliferation. On the other hand, activation of IGF-II/IGF1R dependent pathways could be also triggered by ERα. Accordingly, ERα knock-down was more effective than an IGF1R antibody in controlling H295R cell proliferation [15]. However, the molecular mechanisms involved in IGF-II-induced ERα phosphorylation and in E2/ERα activation of IGF-II/IGF1R-dependent pathways in ACC are not completely clear.

A large number of studies highlighted that, in cancer cells, ER coregulators play a critical role in hormonal responsiveness and tumor progression [16]. PELP1/MNAR is a novel ER coactivator that exerts an essential role in ER’s actions and its expression is deregulated in hormone driven cancers. PELP1 appears to function as a scaffolding protein, coupling ER with several proteins that are involved in oncogenesis, cell proliferation, and metastasis, such as growth factors [17,18,19,20,21]. These regulatory interactions have important functional implications in the cross-talk of estrogen receptor and growth factors signaling [22,23]. Taking into account all of these observations, the main purpose of this study was to define if PELP1 is expressed in ACC and is able to play a role in ACC growth by promoting cross-talk between ERα and IGF1R.

## 2. Materials and Methods

Cell culture and tissues: H295R cells were obtained from Dr. Antonio Stigliano (Department of Clinical and Molecular Medicine, Sant’Andrea Hospital, Faculty of Medicine and Psychology, Rome, Italy) [24] and cultured in Dulbecco’s modified Eagle’s medium/Ham’s F12 DMEM/F12 (Sigma-Aldrich, Milano, Italy) supplemented with 1% ITS Liquid Media Supplement (Sigma), 10% fetal bovine serum, 1% glutamine, 2% penicillin/streptomycin (Sigma) (complete medium), at 37 °C in an atmosphere of humidified air containing 5% CO_2_. Cells were subcultured for 48 h in complete medium on 100 mm dishes for IP assays (5 × 10^6^ cells/plate), 60 mm dishes for protein extraction (600 × 10^3^ cells/plate), and 24-well culture dishes for proliferation experiments (50 × 10^3^ cells/well).

Prior to experiments, cells were starved overnight in DMEM/F-12 medium containing only antibiotics. Cells were treated with 17β-estradiol (E2) (100 nM) (Sigma) and IGF-II (100 ng/mL) (Sigma). Adrenocortical tumors, removed at surgery, and normal adrenal cortex, macroscopically dissected from adrenal glands of kidney donors, were collected at the hospital-based Divisions of the University of Padova (Padova, Italy). Tissue samples were obtained with the approval of local ethics committees and consent from patients (Project Tissue Biobank Resource n. 130) in accordance with the Declaration of Helsinki.

Diagnosis of malignancy was performed according to the histopathological criteria proposed by Weiss et al. [25] and modified by Aubert et al. [26]. Patients’ clinical data related to ACC samples included in this study are shown in Table 1. Sample C6 belonged to a patient who quit mitotane treatment after six months due to severe gastrointestinal side effects. Patients C1 and C2 received chemotherapy following EAP protocol (etoposide, doxorubicin, and cisplatin) plus mitotane [27].

Western blot analysis: H295R cells, from normal or cancer adrenal tissue samples were lyzed in ice-cold radioimmunoprecipitation assay buffer containing protease inhibitors (20 mmol/L Tris, 150 mmol/L NaCl, 1% Igepal, 0.5% sodium deoxycholate, 1 mmol/L EDTA, 0.1% SDS, 1 mmol/L phenylmethylsulfonyl fluoride, 0.15 units/mL aprotinin, and 10 μmol/L leupeptin). Protein content was determined by the Bradford method. Fifty µg of protein were separated on an 11% SDS-polyacrylamide gel and then electroblotted onto a nitrocellulose membrane.

Blots were incubated overnight at 4 °C with specific antibodies: (a) anti-IGF1Rβ antibody (C-20) (1:500; Santa Cruz Biotechnology, Santa Cruz, CA, USA); (b) anti-pERK1/2 antibody (T202/Y204) (1:1000; Cell Signaling Technology, Bervely, MA, USA); (c) anti-ERK1/2 antibody (1:1000; Cell Signaling Technology); (d) anti-CCND1 antibody (3H2043) (1:1000; Santa Cruz Biotechnology); (e) anti-ERα (F-10) antibody (1:1000; Santa Cruz Biotechnology); (f) anti-c-Src antibody (1:500; Santa Cruz Biotechnology); (g) anti-PELP1/NMAR antibody (1:10,000; Bethyl Laboratories Inc. Montgomery, TX, USA). Membranes were incubated with horseradish peroxidase (HRP)-conjugated secondary antibodies (Amersham Pharmacia Biotech, Piscataway, NJ, USA), and immunoreactive bands were visualized with the enhanced chemiluminescence Western blotting detection system (Amersham). To assure equal loading of proteins, membranes were stripped and incubated overnight with glyceraldehyde-3-phosphate dehydrogenase (GAPDH) antibody (FL-335) (1:2000; Santa Cruz Biotechnology).

MTT assay and [3H] thymidine incorporation: A3-(4,5-Dimethylthiaoly)-2,5-diphenyltetrazolium bromide (MTT) assay, as well as thymidine incorporation, was performed to detect cell proliferation. At the end of each treatment, fresh MTT (Sigma), resuspended in PBS, was added to each well (final concentration 0.33 mg/mL). After 2 h incubation, cells were lyzed in dimethylsulfoxide (Sigma). Each experiment was performed in triplicate and the OD was measured at 570 nm in a monochromator-based multi-mode microplate reader (Synergy H1 Hybrid Reader, BioTek, Germany, Europe). [3H] thymidine incorporation was performed as described in Casaburi et al. [28]. Briefly, H295R as well as 24 h PELP1 silenced H295R cells were untreated or treated with E2 or IGF-II for an additional 48 h. For the last 6 h, [3H] thymidine (1 μCi/mL) was added to the culture medium. After rinsing with PBS, cells were washed once with 10% and three times with 5% trichloroacetic acid. Cells were lyzed by adding 0.1 N NaOH and then incubated for 30 min at 37 °C. Thymidine incorporation was determined by scintillation counting.

RNA interference: PELP1 small interfering RNA (siRNA) and nontargeting siRNA (scrambled or control siRNA) were purchased from Dharmacon (ON-TARGET plus siRNA Human PELP1 cat # J-004463-06 50 nmol) (Invitrogen, Carlsbad, CA, USA). Cells were plated into 60 mm dishes for protein extraction and into 24-well plates for proliferation assay. siRNAs were transfected to a final concentration of 100 or 200 nM using the DharmaFECT transfection Reagent (cat # T-2001-002), according to the manufacturer’s recommendations (Dharmacon, CO, USA). PELP1 knockdown was checked by Western analysis on proteins extracted from cells transfected for 24 h.

Immunoprecipitation assay: H295R cells were lyzed in ice-cold radioimmunoprecipitation assay buffer (see Western blot analysis). The protein content was determined by the Bradford method and 500 μg of protein lysates were first precleared with 10 μL of protein A agarose beads (Santa Cruz) for 1 h. Then precleared cell lysates were incubated over night with primary anti-PELP1 antibody together with 20 μL of protein A agarose beads at 4 °C in HNTG buffer (20 mM HEPES pH 7.5, 150 mM NaCl, 0.1% Triton X-100, 10% glycerol, 0.1 mM Na_3_VO_4_). Negative control samples were obtained by replacing anti-PELP1 antibody with normal rabbit IgG. Immunoprecipitated proteins were washed three times with HNTG buffer, separated on an 11% polyacrylamide denaturing gel, analyzed by WB and visualized by ECL (Amersham).

Data analysis and statistical methods: All experiments were performed at least three times. Data were expressed as mean values ± standard deviation (SD), statistical significance between control and treated samples were analyzed using GraphPad Prism 5.0 (GraphPad Software, Inc.; La Jolla, CA, USA) software. Control and treated groups were compared using the analysis of variance (ANOVA). A comparison of individual treatments was also performed, using Student’s *t*-test. Significance was defined as *p* < 0.05.

## 3. Results

### 3.1. PELP1 Is Expressed in Human ACC Samples and in H295R Cells

**Figure 1:**
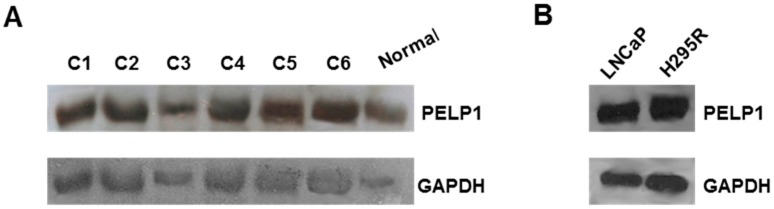
PELP1 expression in human tissues of ACC and H295R cells. (**A**) Western blot analysis of PELP1 was performed on 50 μg of total protein extracted from normal human adrenal tissues (normal) and ACCs (C1–C6); (**B**) Western analysis of PELP1 was performed on 50 μg of total protein extracted from LNCaP and H295R cells. GAPDH was used as a loading control. Results are representative of three different experiments.

We first examined PELP1 expression in normal human adrenal tissue, six different ACC samples, and the H295R cell line. Using Western blot analysis we showed that PELP1 is expressed in normal and ACC samples (Figure 1A), as well as in H295R cells (Figure 1B) with a similar expression pattern to that of prostate carcinoma cell line LNCaP, which was used as a positive control [29].

It is worth noting that differences in PELP1 expression levels were not seen among the ACC samples, despite the different associated chemotherapeutic protocols (Table 1).

### 3.2. PELP1 Is Recruited to Form a Multiprotein Complex in H295R Cells after E2 and IGF-II Treatment

**Figure 2:**
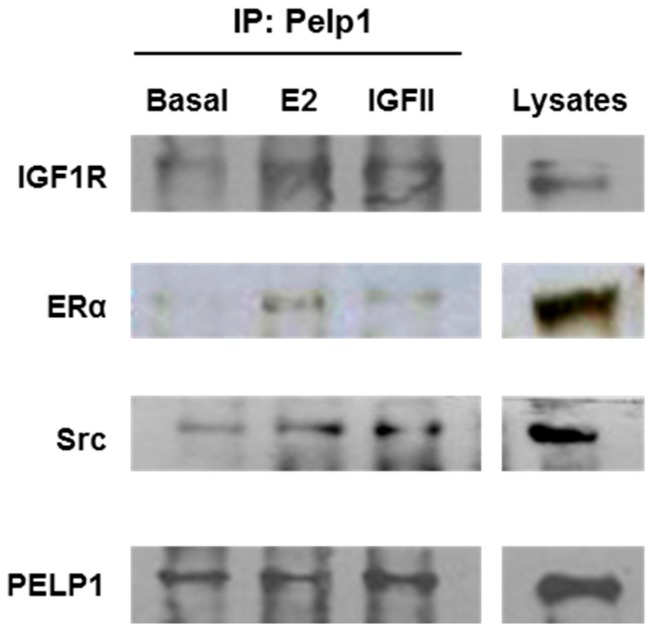
PELP1 is recruited to form a multiprotein complex in H295R cells after treatment with E2 and IGF-II. H295R cells were treated for 10 min with E2 (100 nM) or IGF-II (100 ng/mL). Total protein extract (500 μg) was immunoprecipitated with 1 μg of anti-PELP1 antibody. The samples were immunoblotted for IGF1R, ERα, and c-Src. Protein expression for each sample was normalized to PELP1 content. Results are representative of three independent experiments.

In order to establish a role for PELP1 as a scaffold protein able to connect rapid estrogen-dependent and IGF-II-dependent signaling, we used an anti-PELP1 antibody to immunoprecipitate protein lysates from H295R cells treated for 10 min with E2 or IGF-II. We observed that both treatments rapidly induced the formation of a multiprotein complex in which we revealed the interaction of PELP1with IGFIR, ERα, and c-Src (Figure 2).

### 3.3. PELP1 Knockdown Decreases ERK1/2 Phosphorylation in H295R Cells

**Figure 3:**
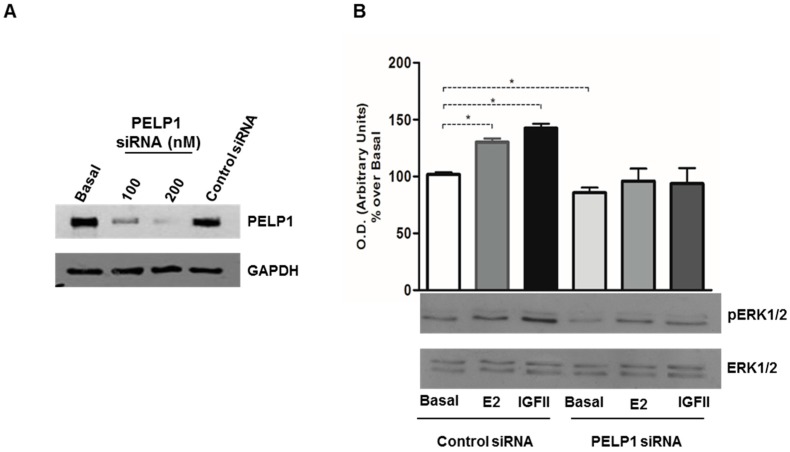
PELP1 knockdown decreases ERK1/2 phosphorylation. (**A**) H295R cells were transfected with PELP1 siRNA (100 nM and 200 nM) or a non-targeting siRNA (control siRNA) for 24 h. Western blot analyses of PELP1 were performed on 50 μg of total protein; (**B**) H295R cells were transfected with control siRNA or PELP1 siRNA. After 24 h cells were treated for 10 min with E2 (100 nM) or IGF-II (100 ng/mL). Western blot analyses of PELP1 were performed on 10 μg of total protein. Results are representative of three independent experiments. GAPDH and ERK1/2 were used as a loading control; upper panel graph represents mean of pERK1/2 optical density (O.D.) from three independent experiments with similar results normalized to ERK1/2 content (* *p* < 0.001 compared to untreated control sample (basal) assumed as 100).

The aim of the next set of experiments was to determine if PELP1 plays a role in rapid ERK1/2 activation induced by E2 and IGF-II. First we tested different concentrations (100 and 200 nM) of a specific siRNA and the reduced PELP1 expression was observed by Western blot analysis (Figure 3A). With the basis of Western blot results, we chose 200 nM as the best siRNA concentration to reduce PELP1 expression in all subsequent experiments.

Next H295R cells were transfected for 24 h with scrambled or siRNA for PELP1 and then treated for 10 min with E2 or IGF-II. In the presence of scrambled siRNA, E2 and IGF-II retained their ability to increase ERK phosphorylation, while in the presence of a reduced PELP1 protein expression the E2- and IGF-II-dependent ERK1/2 activation was decreased (Figure 3B). These data indicate that, in H295R cells, the formation of a multiprotein complex containing PELP1 is required to allow rapid MAPK activation induced by E2 and IGF-II treatment.

### 3.4. PELP1 Knockdown Decreases IGFIR Expression in H295R Cells

**Figure 4:**
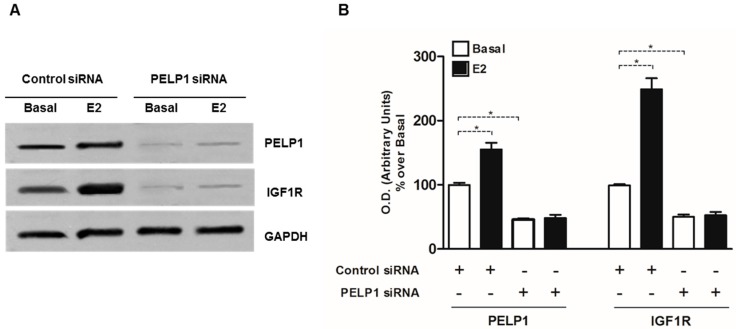
PELP1 knockdown decreases IGF1R expression in H295R cells. (**A**) H295R cells were transfected with PELP1 siRNA or a non-targeting siRNA (control siRNA) for 24 h. After transfection cells were treated for 24 h with E2 (100 nM). Western blot analyses of PELP1 and IGF1R were performed on 50 μg of total protein. Results are representative of three independent experiments. GAPDH was used as a loading control; (**B**) The graph represents mean of PELP1 and IGF1R optical densities (O.D.) from three independent experiments with similar results normalized to GAPDH content (* *p* < 0.001 compared to untreated control sample (Basal) assumed as 100).

Starting from our previous observation that in H295R cells estrogens can modulate IGF1R expression [15], we wanted to investigate the effect of PELP1 silencing on E2-induced IGF1R expression. We confirmed our previous data on the ability of E2 to increase IGF1R expression and we also observed that PELP1 is required for this event. In fact, the ability of E2 to increase IGF-1R protein expression in PELP1 silenced H295R cells was no longer detectable. Importantly, PELP1 silencing reduced IGF1R expression, even in basal conditions (Figure 4).

### 3.5. PELP1 Knockdown Decreases Cyclin D1 Expression in H295R Cells

**Figure 5:**
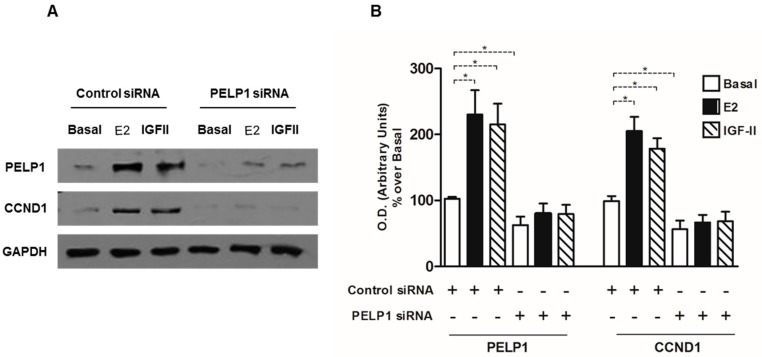
PELP1 knockdown decreases Cyclin D1 expression in H295R cells. Cells were transfected with PELP1 siRNA or a non-targeting siRNA (control siRNA) for 24 h. After transfection cells were treated for 24 h with E2 (100 nM) and IGF-II (100 ng/mL). Western blot analyses of Cyclin D1 were performed on 50 μg of total protein. Results are representative of at least three independent experiments. GAPDH was used as a loading control; the right panel graph represents mean of Cyclin D1 optical density (O.D.) from three independent experiments with similar results normalized to GAPDH content (* *p* < 0.001 compared to untreated control sample (Basal) assumed as 100).

Our previous study indicated that ACC cell proliferation in response to E2 and IGF-II relies on the activation of Cyclin D1 [15]. For this reason, we evaluated Cyclin D1 expression in H295R cells after PELP1gene silencing. Our experiments showed that in H295R cells the reduced PELP1 expression abrogated the increase in Cyclin D1 expression induced by E2 and IGF-II treatment. It is worth noting that, in basal condition also, the Cyclin D1 expression is lowered in the presence of silenced PELP-1 expression (Figure 5).

### 3.6. PELP1 Knockdown Reduces Proliferation of H295R Cells

**Figure 6:**
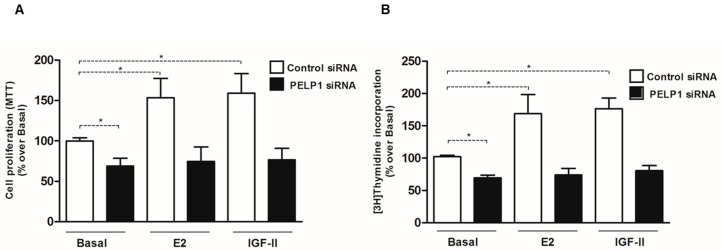
PELP1 knockdown reduces cell proliferation in H295R. (**A**,**B**), H295R cells were transfected with PELP1 siRNA or a non-targeting siRNA (control siRNA). Twenty-four hours after silencing, cells were left untreated (Basal) or treated for an additional 48 h with IGF-II (100 ng/mL) or E2 (100 nM). (* *p* < 0.0001 compared with Basal). At the end of the experiment, cells were incubated with MTT (**A**) or [3H] thymidine (**B**) as described in Materials and Methods.

Considering that PELP1 silencing was able to reduce the expression of genes involved in E2- and IGF-II-dependent H295R cell growth, we investigated the effects of PELP1 silencing on H295R cell proliferation. As shown in Figure 6A,B, PELP1 silencing in H295R cells significantly reduced the ability of both E2 and IGF-II to induce cell proliferation as assessed by both MTT assay and [3H] thymidine incorporation. Moreover, according to the above results where the expression of IGF-1R and Cyclin D1 was reduced, basal cell proliferation was also affected in untreated PELP1-silenced H295R cells.

## 4. Discussion

The aim of this study was to determine if PELP1 is expressed in ACC and whether or not it may play a role in promoting the interaction between ERα and IGF1R, allowing the activation of signaling pathways controlling ACC cell growth. First, we showed that PELP1 is expressed in H295R cells, as well as in normal adrenal and ACC tissues. We only aimed to assess if PELP1 was expressed in normal and tumor adrenal samples without indicating any difference in the expression levels since a limited number of samples were available. However, the data obtained support the idea of investigating PELP1 expression in a larger number of ACC samples in order to better determine if PELP1 gene expression is altered in ACCs. Of note, PELP1 expression was found to be deregulated in several cancers [30], and can be upregulated by estrogens and differentially regulated by selective estrogen receptor modulators [16].

It has been demonstrated that PELP1 participates in ER cytoplasmic and membrane mediated signaling events by coupling ER with several cytosolic kinases acting as a scaffolding protein and facilitating the activation of ER-mediated non-genomic signaling [20]. PELP1 modulates the interaction of ERs with c-Src, stimulating c-Src enzymatic activity, leading to the activation of the mitogen activated protein kinase (MAPK) pathway [31]. Moreover, growth factors promote tyrosine and serine phosphorylation of PELP1 [19] which can directly interact with several growth factor receptors in particular EGFR and HER2 [18,19,20,21]. Such regulatory interactions of PELP1 have important functional implications in the cross-talk of estrogen receptor and growth factor signaling [18].

In H295R cells, we demonstrated that 10 min of treatment with E2 or IGF-II allowed the formation of a multiprotein complex consisting of PELP1, IGF1R, ERα, and c-Src. Moreover, while E2 and IGF-II were able to induce rapid ERK1/2 activation, this effect was lost after PELP1 silencing. These data indicate PELP1 as a key player in both E2- and IGF-II-dependent ERK1/2 phosphorylation in H295R cells. Similar mechanisms have been revealed in breast cancer cells where PELP1 has been shown to be a critical mediator of estrogen-induced MAPK activation via c-Src. In fact, upon E2 stimulation, ERα induces rapid (5–10 min) activation of MAPK in a c-Src dependent manner [32]. Mutation of the PELP1 PXXP c-Src interaction site abolished estrogen-induced MAPK activation and ER transcriptional activity. ER is also able to interact with c-Src, further increasing signaling efficiency to MAPK [33]. The PELP1/ER/c-Src signaling axis has also been shown to include growth factor receptors (EGFR, HER2) and the stimulation of PI3K/AKT and integrin-linked kinase 1 (ILK1) pathways [19,34,35,36]. Here, we demonstrated that PELP1 physically interacts with the IGF1R and this interaction is a required molecular event for IGF-II-mediated ERK1/2 phosphorylation. Mechanistic studies demonstrated that overexpression of a mutated form of PELP1 that lacks the nuclear localization signal was able to drive MAPK signaling and constitutive AKT activation in unstimulated breast cancer cells, resulting in increased phosphorylation of ERα at Serine 118 and Serine 167 [19]. Starting from these observations, we hypothesized that PELP1 could also have a role in IGF-II-dependent ERα phosphorylation that we previously demonstrated in H295R cells [15]. On the other hand, E2 is able to modulate IGF1R expression as a consequence of increased cAMP-responsive element binding (CREB) protein activation and binding to the IGF1R promoter [15]. Here, we demonstrated that PELP1 silencing is able to inhibit both basal and E2-induced IGF1R protein expression.

A further demonstration of PELP1 involvement in pathways regulating ACC cell growth came from the observation that PELP1 gene silencing was able to decrease basal and abrogate E2- and IGF-II-dependent expression of Cyclin D1. Accordingly, reduced H295R cell growth was also detected after PELP1 gene silencing. Importantly, silencing of PELP1 using a liposomal formulation to vehicle PELP1 siRNA in vivo was shown to be significantly effective in reducing the growth of an orthotopic model of ER positive breast cancer [37], suggesting that targeting this protein is feasible in vivo and could have promising therapeutic effects. Further studies will be required to define if PELP1 has any genomic effect in ACC cells. So far, our data suggest that the main role played by PELP1 in controlling ACC cell growth is by coupling ERα with IGF1R and c-Src, working as a scaffolding protein and facilitating the activation of ER-nongenomic signaling and IGF1R-dependent pathways.

## 5. Conclusions

In conclusion, we demonstrated for the first time that PELP1 is expressed in ACC tissues and H295R cells. Moreover, we have shown that E2 and IGF-II are able to induce the formation of a multiprotein complex consisting of PELP1, IGF1R, ERα, and c-Src and are involved in the rapid activation of ERK1/2. The important role played by the PELP1/ER/IGF1R/c-Src signaling axis in promoting both E2- and IGF-II-dependent ACC cell proliferation suggests that further studies are needed to consider PELP1 as a new target for the therapy of ACCs.

## Figures and Tables

**Table 1:** Clinical data of the six ACC patients analyzed in this study.

Sample ID	Age (Years)	Gender	Stage at Surgery	Syndrome	Weiss Score	Size (cm)	Outcome
C1	41	M	IV	Cushing	9	16	Died, 1 year
C2	17	F	IV	Cushing	9	14	Died, 18 months
C3	43	F	III	None	4	9	Died, 8 years
C4	46	M	III	None	3	18	Remission, 7 years
C5	47	M	IV	Cushing	9	14	Died, 1 year
C6	57	M	II	Subclinical Cushing	5	14	Remission, 4 years
